# The mind–body connection in women’s reproductive health: how psychological stress shapes gut and ovarian function - a narrative review

**DOI:** 10.3389/fgstr.2026.1854862

**Published:** 2026-10-12

**Authors:** Arpitha Yachanahalli Purushotham, Borehalli Mayegowda Shilpa, Divya Sundaram

**Affiliations:** School of Psychological Sciences, CHRIST University, Bengaluru, India

**Keywords:** HPA-axis, psychological stress, neuroendocrinology, reproduction, microbiome, mindbody, cognitive behavioural therapy

## Abstract

Psychological stress plays a critical role in modulating women’s reproductive health through integrated neuroendocrine, immune, and microbiome-mediated pathways. Activation of the hypothalamic–pituitary–adrenal (HPA) axis leads to elevated secretion of corticotropin-releasing hormone (CRH), adrenocorticotropic hormone (ACTH), and cortisol, which suppress hypothalamic gonadotropin-releasing hormone (GnRH) pulsatility, thereby disrupting the hypothalamic–pituitary–gonadal (HPG) axis. This results in reduced secretion of luteinising hormone (LH) and follicle-stimulating hormone (FSH), impairing folliculogenesis, ovulation, and menstrual cyclicity. Chronic stress further induces oxidative stress, mitochondrial dysfunction, and apoptosis in ovarian cells, thereby reducing oocyte competence and diminishing ovarian reserve. Additionally, stress-induced gut microbiota dysbiosis alters the composition of key microbial taxa, including *Lactobacillus, Bifidobacterium, and Clostridium*, disrupting the estrobolome and impairing oestrogen metabolism. This dysregulation increases intestinal permeability and promotes systemic inflammation, as evidenced by biomarkers including interleukin-6 (IL-6), tumour necrosis factor-alpha (TNF-α), and C-reactive protein (CRP), which adversely affect endometrial receptivity and implantation success. Emerging evidence also indicates that microbial metabolites such as short-chain fatty acids (SCFAs), tryptophan metabolites, and serotonin play a crucial role in modulating neuroendocrine signalling and reproductive outcomes. Despite growing evidence, the lack of standardised biomarkers and well-designed clinical trials limits translation into clinical practice. Future research should focus on integrative, personalised therapeutic approaches combining psychological interventions, lifestyle modifications, and microbiome-targeted therapies to improve reproductive outcomes and overall well-being.

## 1 Introduction

Women’s reproductive health represents a highly regulated and dynamic biological system characterised by the integration of endocrine signalling, ovarian physiology, and systemic homeostatic mechanisms. The hypothalamic–pituitary–gonadal (HPG) axis plays a central role in coordinating hormonal cascades that govern folliculogenesis, ovulation, and menstrual cyclicity. Concurrently, accumulating evidence underscores the significance of the human microbiome as a critical determinant of physiological equilibrium, extending beyond gastrointestinal function to influence metabolic, immunological, and reproductive processes. The host-associated microbial ecosystem contributes to maintaining systemic homeostasis through metabolic and signalling pathways Kennedy et al. (1). Similarly, the Human Microbiome Project Consortium (2) emphasised that both the compositional diversity and functional capacity of microbial communities are essential for sustaining normal physiological states. In recent years, scientific inquiry has increasingly focused on the integrative interactions between endogenous biological systems and exogenous psychological and environmental factors, particularly in the context of reproductive outcomes (3).

The conceptual framework of mind–body integration provides a mechanistic basis for understanding how psychological states modulate physiological function. Central to this paradigm is the microbiota–gut–brain axis, a bidirectional communication network linking central nervous system activity with gastrointestinal and microbial dynamics. This axis operates through coordinated neural, endocrine, and immune signalling pathways, enabling continuous feedback between emotional–cognitive centres and peripheral organ systems. Marano et al. (4), further demonstrated that this complex communication network is integral to maintaining homeostasis, while its dysregulation is implicated in the pathogenesis of multiple systemic disorders. Grenham et al. (5). Within this context, reproductive health must be conceptualised as a product of both biological regulation and psychological modulation, necessitating an interdisciplinary approach that integrates neuroendocrinology, immunology, and microbiome science.

Psychological stress has emerged as a critical modulatory factor influencing reproductive health through its effects on neuroendocrine and microbial homeostasis. Chronic stress activates the hypothalamic–pituitary–adrenal (HPA) axis, resulting in elevated glucocorticoid secretion, particularly cortisol, which can disrupt gonadotropin-releasing hormone (GnRH) pulsatility and downstream reproductive hormone synthesis. Stress-induced alterations in gut microbiota composition can significantly influence neuroendocrine signalling and immune responses Foster et al. (6).

Chronic stress contributes to microbial dysbiosis, which in turn perturbs ovarian function and hormonal balance. Ma et al. (7). These perturbations are associated with a spectrum of reproductive pathologies, including infertility, menstrual irregularities, and metabolic dysfunctions. The link between microbiome imbalance and systemic inflammation, suggests that pro-inflammatory states may serve as a mechanistic bridge connecting stress and reproductive dysfunction. Hamjane et al. (8) and Garg et al. (9).

A unique microbiota has been identified in the female reproductive tract, with different parts of the system featuring unique communities. Lactobacillus species are the primary microorganisms of the vaginal microbiome and are found in high numbers in healthy reproductive-age women, producing lactic acid to keep vaginal pH low and protect against pathogenic microorganisms. The microbial composition of the cervix, endometrium, fallopian tubes and ovaries is less well established and typically has a smaller microbial biomass. This disturbance of the microbial balance can make a woman more susceptible to bacterial vaginosis, pelvic inflammatory disease, infertility, endometriosis, polycystic ovary syndrome, implantation failure, and complications with pregnancy. Hence, the gynaecological microflora should be considered as a living ecosystem, which is regulated by hormonal status, the phase of the menstrual cycle, sexual activity, exposure to medications, ethnicity, diet, immune function and psychological stress.

There is also increasing evidence of a connection between reproductive-tract dysbiosis, endometrial inflammation and carcinogenesis via immune response changes epithelial-barrier dysfunction, microbial metabolite production, and sustained inflammatory signalling. Some differences in the composition of endometrial cancer patients’ microbiota have been observed from healthy women, though the findings so far are still conflicting and not conclusive of causation (10). The findings extend the clinical relevance of microbiome studies beyond fertility and non-cancerous reproductive diseases and suggest that the interplay between the gut microbiome, vaginal microbiome, endometrial environment, sex hormones and psychological factors play a role in a woman’s reproductive health.

This narrative review examines how psychological stress may influence female reproductive health through interactions among the HPA and HPG axes, gut and reproductive-tract microbiota, inflammatory pathways, and ovarian function. Particular attention is given to the stress–gut–ovary axis and its possible relevance to menstrual disturbances, Polycystic ovary syndrome (PCOS), endometriosis, infertility, and assisted reproductive outcomes. The review also evaluates current therapeutic approaches, limitations in the available evidence, and priorities for future research.

Female reproductive health and fertility are mainly determined by the microbiome as they lodge various types of beneficial as well as infectious microbes. The female fertility and gut- reproductive tract microbiome axis are interconnected to each other. Poor gut health can alter the gut microbiome level in the female reproductive tract, which can be influenced by various lifestyle factors such as high-fat diets, obesity, stress, medications, inflammatory diseases, and poor exercise (11, 12), causing dysbiosis. A few anaerobic microbes like *Atopobium*, *Prevotella*, and *Sneathia* found in the vaginal microbiome are responsible for dysbiosis leading to the clinical condition bacterial vaginosis. According to a few recent studies, a healthy female reproductive tract needs an environment rich in the Lactobacillus microbiome, which produces lactic acid, hydrogen peroxide, and antimicrobial compounds that help maintain a low pH level and stop the growth of excessive anaerobic bacteria (11, 12).

Reduction in healthy microbiome levels will cause decline in the short-chain fatty acids (SCFAs) like butyrate, acetate, propionate productions, which in turn causes weak intestinal barrier, through which bacterial lipopolysaccharide and other microbial molecules will start entering the blood stream causing leaky gut or intestinal permeability (11, 12). Decreased vaginal secretion of lactic acid and overproduction of hydrogen peroxide will increase pH levels in the vagina leading to overgrowth of anaerobic bacteria also causing bacterial vaginosis along with dysbiosis and inflammation (11, 12). The reproductive tract microbiome plays a pivotal role in female fertility, with *Lactobacillus*-dominated communities maintaining a protective, low pH environment. A study also reported the occurrence of degradation of plastic by uterine microbiota like *Bacillus pseudofirmus* and *Stenotrophomonas rhizophila*, which may be because of environmental pollutant which may cause endometrium related cancer and may also interfere with fertility (10). This inflammation might also extend to the cervix and endometrium areas in the female reproductive tract which can lead to chronic endometritis, which causes changes in the inflammatory cytokinesis like interleukin-6 (IL-6), interleukin-1b (IL-1b), and tumour necrosis factor-alpha (TNF-α) and affect the embryo implantation by causing the endometrium to shift away from the immune balanced state which is very much required during implantation (11). Beneficial reproductive tract bacteria increase proinflammatory cytokines and systemic inflammation by activating the innate immune receptors (12) which disturb the insulinsensitivity, lipid metabolism, hormonal regulations, or the HPA axis signalling causing an indirect effect on the fertility in women (11). Especially conditions like PCOS in females, can lead to higher testosterone, hyperandrogenism, irregular menstrual cycles and oligo-ovulation affecting the natural conception process (11).

Finally, it leads to impairment in the embryo endometrium communication, disturbances during the implantation window, weakening of trophoblast invasion, reduced implantation rates, increase clinical pregnancy and live birth rates especially in conditions with IVF and multiple failures of implantation. Even if pregnancy is achieved during these conditions, there to an increased risk of ascending infection, preterm labour, membrane rupture or even miscarriage (11, 12). The gut microbiome can act as a modifiable factor that can indirectly affect the reproductive tract and fertility rates. Therefore, by improving diet and fibre intakes, addressing metabolic dysfunctions, increased lactobacillus microbiome and by reducing the intake of medication using probiotics, antibiotics, metformin, or even having microbiota based therapies could potentially help in reducing the inflammations and improving the reproductive conditions as well as fertility in females (13).

## 2 Methods

### 2.1 Review design and reporting approach

This study was designed as a narrative review to provide an integrative synthesis of the evidence concerning psychological stress, gut and gynaecological microbiota, ovarian function, and women’s reproductive health. A narrative approach was selected because the topic includes heterogeneous evidence from human observational studies, clinical investigations, experimental studies, mechanistic research, and previous reviews. The review was conducted and reported according to the principles of the Scale for the Assessment of Narrative Review Articles (SANRA), including justification of the review’s importance, a clear statement of its aims, description of the literature-search approach, appropriate scientific referencing, balanced presentation of evidence, and relevant endpoint data. Relevant reporting resources available through the EQUATOR Network were also consulted to improve transparency and completeness.

### 2.2 Information sources and search strategy

A structured literature search was undertaken using PubMed/MEDLINE, Scopus, Web of Science, and Google Scholar. The search focused primarily on peer-reviewed literature concerning psychological stress, neuroendocrine regulation, microbiota, ovarian physiology, and reproductive disorders. Search terms were used individually and in appropriate combinations, including “psychological stress,” “chronic stress,” “female reproductive health,” “gut microbiota,” “vaginal microbiome,” “endometrial microbiome,” “gynaecological microbiota,” “gut–brain–ovary axis,” “HPA axis,” “HPG axis,” “ovarian function,” “estrobolome,” “oestrogen metabolism,” “polycystic ovary syndrome,” “endometriosis,” “infertility,” and “assisted reproductive technology.” Reference lists of relevant articles were also screened to identify additional eligible publications.

### 2.3 Eligibility criteria

Studies were considered eligible when they examined at least one relationship among psychological stress, neuroendocrine activity, gastrointestinal or reproductive-tract microbiota, inflammation, oestrogen metabolism, ovarian function, or female reproductive outcomes. Human observational studies, clinical studies, controlled trials, experimental studies, systematic reviews, and high-quality narrative reviews published in English were considered. Articles were excluded if they were unrelated to women’s reproductive health, lacked sufficient scientific relevance, were available only as abstracts, were non-scholarly publications, or substantially duplicated evidence already included from a more complete source.

### 2.4 Study selection and evidence synthesis

Titles and abstracts identified through the literature search were assessed for relevance, followed by examination of the full texts of potentially eligible publications. Information was extracted on study design, population or experimental model, exposure or intervention, microbiological or biological pathway, reproductive outcome, and principal findings. Owing to substantial heterogeneity in study populations, stress-assessment methods, microbiome-sampling procedures, reproductive outcomes, and analytical approaches, statistical meta-analysis was not considered appropriate. The evidence was therefore synthesised narratively and organised into neuroendocrine, ovarian, microbial, immune-inflammatory, clinical, and therapeutic themes. Greater interpretative weight was assigned to human clinical evidence and replicated findings, while evidence derived mainly from animal models or associative microbiome studies was identified as preliminary.

## 3 Neuroendocrine mechanisms of stress

The neuroendocrine response to psychological stress is primarily mediated through the HPA axis, which serves as a fundamental system that controls internal body balance during periods of stress. Stress causes the hypothalamus to release corticotropin-releasing hormone (CRH), which triggers the pituitary gland to produce adrenocorticotropic hormone (ACTH), according to Valsamakis et al. (14). This biochemical sequence culminates in the adrenal cortex producing cortisol. Cortisol functions as the “stress hormone” because it controls how the body handles metabolic, immune, and endocrine responses. Kivimäki et al. (15) showed that persistent HPA axis activity causes multiple body systems to experience functional disturbances, which affect reproductive system functions (**Figure 1**). The figure demonstrates how stress activates the HPA axis, which leads to cortisol production that affects various body systems, such as the reproductive system, the immune system, the metabolic system and the neurological system.

**Figure 1:**
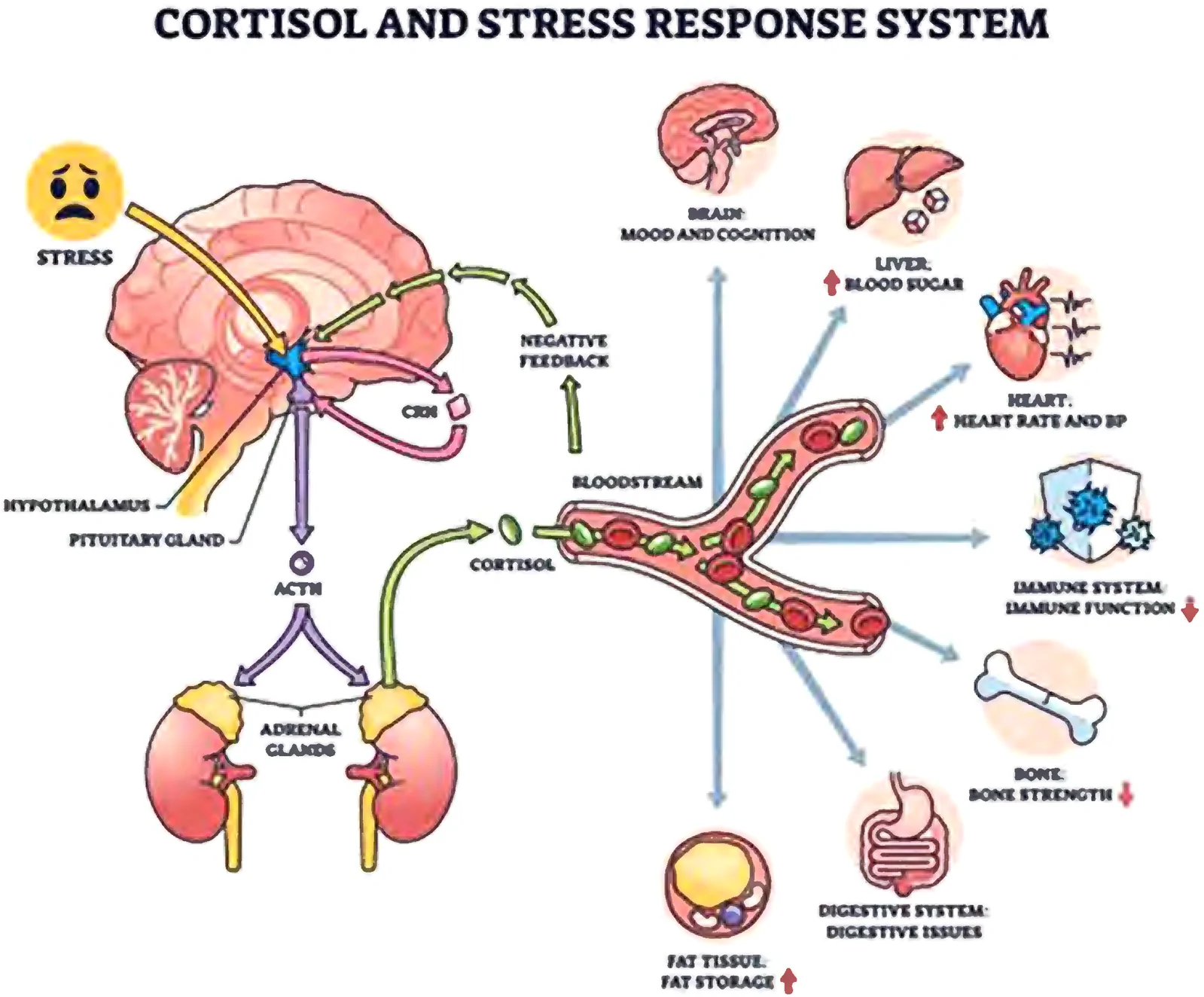
The Hypothalamic–Pituitary–Adrenal (HPA) axis and systemic effects of stress**;** Stress-induced activation of the hypothalamic–pituitary–adrenal (HPA) axis and its inhibitory effects on the hypothalamic–pituitary–gonadal (HPG) axis. Elevated cortisol suppresses gonadotropin-releasing hormone (GnRH) pulsatility, leading to reduced luteinising hormone (LH) and follicle-stimulating hormone (FSH) secretion, thereby impairing folliculogenesis, ovulation, and endometrial receptivity. Chronic hypercortisolaemia further induces systemic inflammation and oxidative stress, compromising ovarian function and oocyte quality. HPA–HPG crosstalk, influenced by gut microbiota and sex-specific neuroendocrine mechanisms, underscores the complex, multifactorial regulation of stress-induced reproductive dysfunction, as supported by recent neuroendocrine and microbiome research.

Cortisol functions as the final product that results from HPA axis activation, and it produces major impacts on essential reproductive hormones, including GnRH, luteinizing hormone (LH) and follicle-stimulating hormone (FSH). The presence of elevated cortisol levels leads to a decrease in GnRH pulsatility, which results in diminished LH and FSH production by the pituitary gland Lawson et al. (16). The hormonal disruption results in negative effects that stop follicles from developing properly, prevent ovulation, and decrease the chances of achieving pregnancy. Schliep et al. (17) found that increased stress levels caused women to experience different ovulation patterns. Stress negatively impacts a woman’s ability to conceive a child due to a woman’s psychological stress of affects her ovulatory function. Stress can lead to an increase's likelihood of anovulation hormonal imbalance and malfunctions. Stress can harm one's most natural of functions, that being fertility. This happens due to the body going through a change in control of reproductive hormones (**Table 1**).

**Table 1:** Effects of psychological stress on neuroendocrine regulation and ovarian function.

Mechanism	Key components affected	Physiological impact	Reproductive outcome	References
HPA Axis Activation	CRH, ACTH, Cortisol	Increased cortisol secretion under stress	Hormonal imbalance	14, 15, 18
HPA–HPG Axis Interaction	GnRH suppression	Reduced LH and FSH secretion	Impaired ovulation	14, 16
Cortisol Effects	Elevated glucocorticoids	Inhibition of GnRH pulsatility	Menstrual irregularities	16, 17
Ovarian Follicular Development	Follicle maturation	Reduced ovarian reserve	Infertility risk	19, 20
Oocyte Quality	Mitochondrial dysfunction, oxidative stress	Poor oocyte competence	Reduced fertilisation success	20, 21
Menstrual Cycle Regulation	Oestrogen, Progesterone imbalance	Irregular cycles (amenorrhea, oligomenorrhea)	Reduced fertility	22
Ovulatory Function	Hormonal dysregulation	Anovulation	Decreased conception probability	17, 23

Psychological stress disrupts reproductive function through the neuroendocrine pathways, with emphasis on HPA axis activation, HPA–HPG axis crosstalk, and glucocorticoid-mediated suppression of GnRH pulsatility. These mechanisms contribute to impaired folliculogenesis, altered steroidogenesis, reduced oocyte competence, menstrual irregularities, and anovulation, ultimately affecting fertility outcomes.

Cortisol-mediated changes affect two different areas: direct hormonal suppression and environmental changes. Albert and Newhouse (24) explained how oestrogen interacts with stress pathways to create mood disorders that subsequently lead to reproductive problems, which indicated that stress can cause endocrine changes that affect female health through the effect on gut microbiome and immune system interactions. Siddiqui et al. (25) The findings demonstrate that stress activates neuroendocrine mechanisms through HPA and HPG axis interaction, which uses cortisol as the primary signalling molecule. This complex network system establishes control over reproductive hormone patterns, which makes stress management crucial for maintaining reproductive health.

Psychological stress significantly alters gut microbiota composition, leading to reduced abundance of beneficial bacteria such as *Lactobacillus* and *Bifidobacterium*, and increased proliferation of opportunistic pathogens such as *Clostridium* species. This dysbiotic state disrupts the estrobolome, a collection of microbial genes involved in oestrogen metabolism, resulting in impaired deconjugation and recirculation of oestrogens. Consequently, altered oestrogen levels affect ovulation, menstrual regulation, and endometrial function. In addition, microbial metabolites such as short-chain fatty acids (butyrate, acetate, propionate), tryptophan-derived metabolites, and serotonin play a key role in regulating neuroendocrine signalling. Reduced SCFA production contributes to increased intestinal permeability “Leading to leaky gut”, facilitating systemic inflammation and immune activation. These changes interfere with ovarian steroidogenesis and follicular development, thereby linking gut dysbiosis directly to reproductive dysfunction (**Figure 2**).

**Figure 2:**
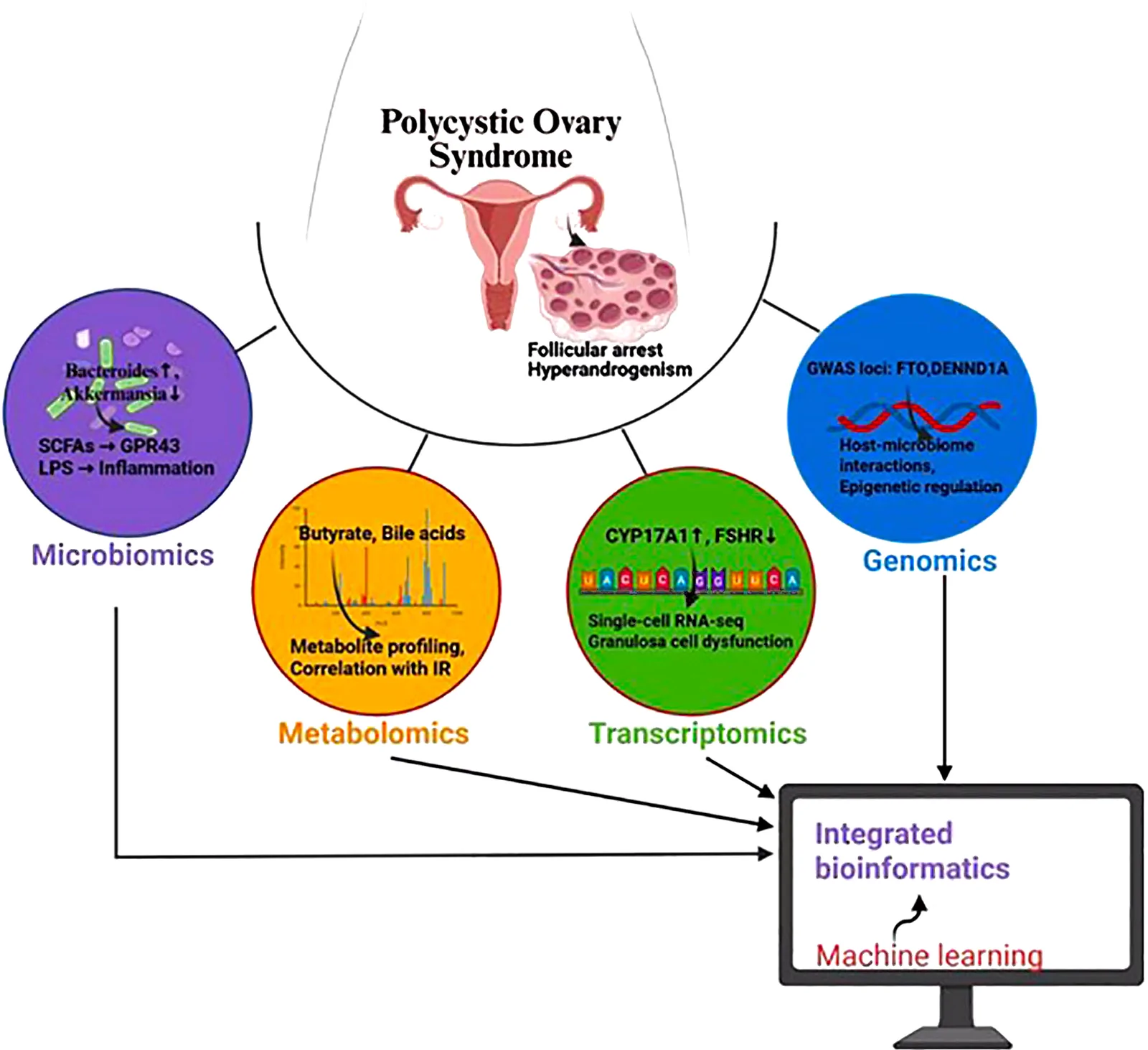
Bidirectional communication between gut microbiota and brain via neural, endocrine, and metabolic pathways. The gut–brain axis, highlighting the bidirectional interactions between the central nervous system and gastrointestinal microbiota. Neural signalling occurs primarily through the vagus nerve, while microbial metabolites, including short-chain fatty acids (SCFAs), and neurotransmitters influence brain function and behaviour. In turn, the brain modulates gut physiology, including motility, secretion, and immune responses. These interconnected pathways demonstrate how gut microbiota and the brain dynamically communicate to regulate physiological homeostasis and stress responses.

## 4 Impact of stress on ovarian function

Psychological stress exerts a profound influence on ovarian function through complex neuroendocrine and physiological pathways. Stress is a main cause of female infertility because it disrupts normal ovarian functions. Rooney et al. (21) The primary biological process includes changes that affect how follicles develop and how ovulation occurs. Chronic stress leads to diminished ovarian reserve because it decreases both the quantity and the quality of developing follicles. Gao et al. (19). Sustained stress exposure speeds up ovarian ageing while it hinders normal folliculogenesis development. Jiang et al. (20). Stress activates primordial follicles before their normal time, which results in their rapid depletion, and this restricts the duration of reproductive capability. Xu et al. (26). It predominantly affects follicular development while it serves as a main cause of menstrualcycle disruptions and hormonal imbalances. People who experience psychological stress have disrupted their body hormones, which need to be balanced for the menstrual cycle to function properly. Bala et al. (22). The body experiences this disruption because stress causes changes in the HPG-hormone system, which leads to irregular patterns of key reproductive hormone release that include oestrogen and progesterone. Women with this condition will experience oligomenorrhea, amenorrhea or anovulatory cycles. Women under chronic stress lead to experience menstrual disturbances, which demonstrate the connection between theemotional state and ability to conceive.

Moreover, stress has a detrimental effect on oocyte quality, which leads to negative impacts on overall fertility results. Elevated stress levels decrease ovarian reserve while also reducing oocyte competence, which serves as a key requirement for successful fertilisation and embryo development Gao et al. (19). Stress creates oxidative and hormonal disturbances, which result in mitochondrial dysfunction which decreases oocytes’ capacity to develop. Jiang et al. (20) Oocyte quality serves as the main factor that determines success in all assisted reproductive technologies. Psychological stress decreases pregnancy rates because it impacts the physiological and behavioural elements that drive human fertility. Rooney et al. (21) showed that psychological stress creates multiple negative effects on ovarian function, which include disruptions to follicular development, hormonal regulation and oocyte quality. The combined effects of these disturbances result in menstrual irregularities, decreased ovarian reserve and diminished fertility potential. Stress management and understanding stress patterns prove vital for maintaining ovarian health and enhancing women’s reproductive success.

## 5 Gut–brain–ovary axis

The gut-brain-ovary axis serves as a fundamental framework for investigating how gastrointestinal microbiota and central nervous system functions interact with female reproductive processes. The microbiota-gut-brain axis functions as a complex communication system that enables two-way information flow through its neural, endocrine and immune signalling pathways Cryan et al. (27). This axis functions to control both gastrointestinal and neurological systems while also extending its effects to reproductive health. The gut microbiota serves as an essential component for maintaining metabolic stability, immune system regulation and hormonal equilibrium, which are necessary for proper ovarian function. The diversity and integrity of gut microbial communities serve as vital factors that determine reproductive health outcomes.

Psychological stress is a major factor capable of disrupting this delicate axis through the induction of gut microbiota dysbiosis. Stress can significantly alter the composition and diversity of gut microbiota, leading to an imbalance between beneficial and pathogenic microorganisms. Dinan and coworkers (28). The dysbiotic state produces multiple effects, which include higher intestinal permeability, increased systemic inflammation and changed neurochemical signalling. Clarke et al. (29) showed that microbial imbalances produce behaviour changes in people while increasing their ability to handle stress, which creates a cycle of worsening psychological and physical health problems. Stress-related dysbiosis produces negative effects on reproductive health because it reduces ovarian function through its effects on immune system activity and hormone regulation, which leads to diminished fertility results. The gut–brain–ovary axis depends on gut microbiota which it helps process oestrogen. The “estrobolome” includes specific bacteria that inhabit the intestine, which use their enzymatic functions to control oestrogen production through deconjugation and reactivation processes. Cryan et al. (27) demonstrated that microbial diversity serves as a crucial factor for maintaining proper oestrogen levels in the bloodstream, which are necessary for regular menstrual cycles and ovulation. The changes in microbial composition will result in different oestrogen metabolism patterns which produce hormonal disorders and reproductive issues. The hormonal changes affect brain activity and stress regulation, which demonstrates the interconnected system that functions along this axis.

## 6 Immune and inflammatory pathways

Psychological stress impacts reproductive health through immune and inflammatory pathways, which serve as its primary biological mediators. Research shows that stress activates immune systems, which produce pro-inflammatory cytokines that include interleukin-6, tumour necrosis factor-alpha and C-reactive protein. According to the changes in gut microbiota composition it directly affects systemic inflammation by impacting the immune system response pathways. The relationship between microbial balance and immune system performance becomes evident through this interaction. Inflammatory mediators function as immune activation indicators while also contributing to the development of chronic diseases, which include reproductive disorders Calder et al. (30) (**Table 2**). The reproductive system shows a strong connection between chronic inflammation and reproductive dysfunction, which occurs due to extended psychological stress. Medzhitov (33) defined inflammation as a protective mechanism that causes tissue destruction and organ system malfunction when it becomes uncontrolled or continues for too long. The reproductive system experiences continual inflammation throughout its processes, which leads to ovarian disturbances and changes in endometrial receptivity affecting hormonal communication methods. Low-grade chronic inflammation causes endocrine and metabolic disorders, which lead to fertility problems. Calder et al. (30) Inflammation caused by gut microbiota dysbiosis increases reproductive health problems through its effect on systemic immune system disturbances. Al Bander et al. (32).

**Table 2:** Role of gut microbiota, immune responses, and stress in reproductive disorders.

Factor	Key elements	Mechanism	Clinical impact	References
Gut Microbiota Dysbiosis	Reduced diversity, pathogenic overgrowth	Altered gut permeability and signalling	Hormonal imbalance	27, 28
Estrobolome Function	Oestrogen-metabolising bacteria	Dysregulated oestrogen recycling	Menstrual disturbances	27, 31
Inflammatory Cytokines	IL-6, TNF-α, CRP	Chronic systemic inflammation	Ovarian dysfunction	30, 32
Immune Dysregulation	Glucocorticoid resistance	Persistent inflammation	Tissue damage in reproductive organs	30, 33
PCOS	Hyperandrogenism, insulin resistance	Stress-induced endocrine disruption	Anovulation, infertility	34, 35
Endometriosis	Ectopic endometrial tissue	Immune + inflammatory activation	Pelvic pain, infertility	36
Infertility (ART/IVF)	Hormonal + psychological stress	Reduced implantation and embryo quality	Lower pregnancy rates	37–39
Gut–Brain Axis	Neural, endocrine signalling	Bidirectional stress feedback	Worsening reproductive dysfunction	27, 40

The stress-mediated reproductive dysfunction is linked with gut microbiota dysbiosis, estrobolome activity, and immune dysregulation mechanistic pathways. It highlights the roles of inflammatory cytokines, glucocorticoid resistance, and neuroendocrine signalling in conditions such as PCOS, endometriosis, and infertility, along with their associated clinical outcomes.

Glucocorticoid resistance serves as another key pathway that connects stress with immune function and reproductive health. Glucocorticoids, including cortisol, function as anti-inflammatory agents because they control immune system activities under typical circumstances. Extended periods of high cortisol levels, which occur during persistent stress, create glucocorticoid resistance because immune cells become less responsive to glucocorticoids. It is vital to aim for inflammation through dietary and lifestyle changes to improve overall health outcomes because chronic, low-grade inflammation is a main underlying cause of many non-communicable diseases. Calder et al. (30) This is because of the complex interactions between immune responses, diet, and lifestyle factors. Though inflammation serves as a fundamental protective mechanism of the immune system; it may lead to disease when it becomes chronic or dysregulated. This underlines the concept that the same inflammatory pathways that safeguard against infection and injury can also promote the advent of metabolic, autoimmune, and degenerative diseases, thereby featuring the dual nature of inflammation as both advantageous and potentially detrimental, contingent upon its regulation. Medzhitov (33).

## 7 Clinical relevance to reproductive disorders

Psychological stress is not considered the sole cause of PCOS, endometriosis, or infertility. Instead, it may modify disease severity, symptom perception, treatment adherence, and quality of life (41, 42). In PCOS, stress-related HPA-axis activation may interact with insulin resistance, hyperandrogenism, obesity, and low-grade inflammation. Gut microbial alterations have also been reported in PCOS, although their causal role and clinical utility remain uncertain (41, 43).

In endometriosis, chronic pain and infertility can substantially increase anxiety, depression, sleep disturbance, and perceived stress. Stress-related immune and inflammatory changes may influence symptom severity; however, evidence that psychological stress initiates endometriosis remains insufficient. The relationship is more appropriately described as bidirectional, with disease symptoms increasing psychological distress and stress potentially amplifying pain and inflammation (41, 42).

Infertility and assisted reproductive treatment are also associated with considerable emotional burden. Stress may affect treatment continuation, health behaviours, and quality of life, but evidence that psychological distress independently reduces IVF success remains inconsistent. In addition, vaginal, endometrial, and gut microbial profiles have been associated with fertility and assisted reproductive outcomes, although their clinical application requires further validation (12, 44, 45). Psychological assessment and support should therefore be offered to improve patient well-being and treatment engagement rather than being presented as a guaranteed method for increasing pregnancy rates (42) (**Figure 3**).

**Figure 3:**
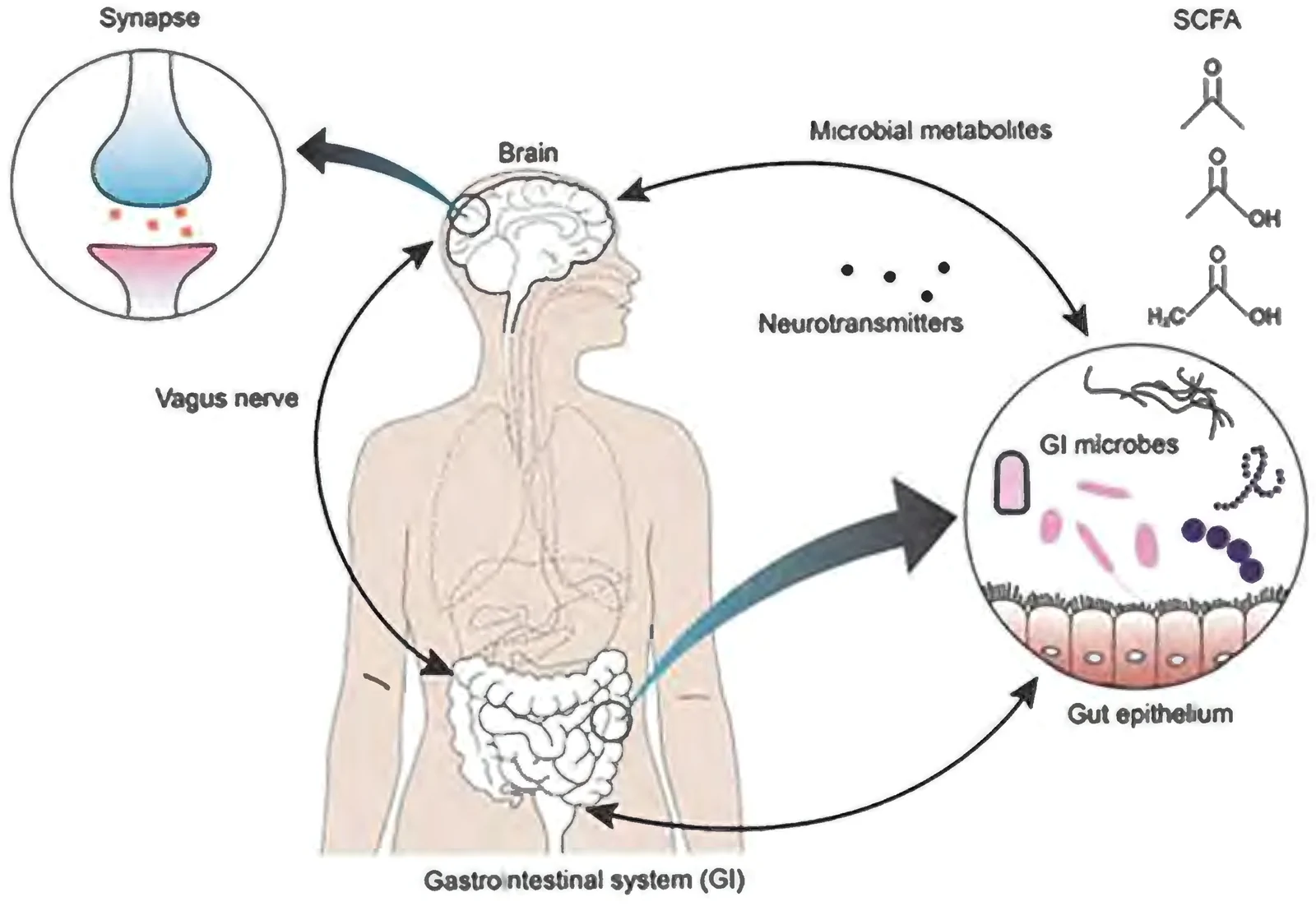
Multi-omics framework for investigating PCOS. Genomic, transcriptomic, metabolomic, and microbiome data may help clarify interactions among microbial dysbiosis, metabolic dysfunction, hyperandrogenism, and follicular arrest. These approaches remain primarily investigational.

## 8 Therapeutic and preventive strategies

Psychological interventions have an important role to play in stress-related reproductive dysfunction. Cognitive Behavioral Therapy (CBT), mindfulness, relaxation training and counselling can help to decrease anxiety, increase emotional resilience and enhance treatment adherence. Stress can disrupt the communication between the HPA–HPG axis, causing anovulation by reducing the pulsatility of GnRH. The HPA axis is also dysregulated in the chronic stress state, which is another reason for integrating psychological care with reproductive therapy (18, 46). Stress-related changes in the microbiome can also influence intestinal permeability, immune function and neurobiological processes, with evidence of stress–gut microbiome bidirectional communication (47, 48).

Complementary support includes lifestyle modification like good nutrition, physical activity, proper sleep, maintaining a healthy weight, and limiting intake of substances, which can support healthy metabolic, microbial, and hormonal processes. Dietary patterns can alter the composition of the gut microbiome and host metabolism (49, 50); and obesity-related metabolic disorders could cause dysbiosis and reproductive dysfunction (51).

Microbially-targeted interventions are under investigation but promising. The vaginal and endometrial microbial profile has been linked to endometrial receptivity, invitro fertilisation (IVF) outcomes and implantation (52, 53). Responses to probiotics, prebiotics, and diet interventions could be stress- or behaviour-modifying, but personal responses need to be evaluated (54, 55). Gut microbes also play a role in immune system development, immune tolerance and inflammatory regulation (56, 57). They also interact with the nervous system which further impacts brain development, behaviour, anxiety, and depression (40, 58). So, psychological care, healthy lifestyle practices and reproductive treatment should be combined, while microbiome-based therapies need more clinical validation.

## 9 Discussion

The extant scientific literature delineates a complex and multifactorial relationship between psychological stress and reproductive dysfunction, mediated through intricate interactions among neuroendocrine, immunological, and microbial pathways. Despite substantial progress, significant translational gaps persist, particularly in the identification and validation of integrated, standardised biomarkers. Current approaches predominantly rely on discrete indicators such as cortisol and GnRH within the HPG- axis, alongside inflammatory mediators including IL-6, TNF-α, and C-reactive protein. However, these biomarkers are often studied in isolation, thereby limiting their diagnostic sensitivity and specificity. Furthermore, emerging evidence implicates gut microbiota-derived metabolites—such as SCFAs and tryptophan derivatives—in modulating neuroendocrine signalling and ovarian function. The absence of a unified biomarker framework integrating these multidimensional parameters significantly constrains clinical applicability and the development of precision diagnostics. Methodologically, the predominance of observational and cross-sectional study designs, as exemplified in prior epidemiological investigations, poses substantial limitations in establishing causal inference. While such studies provide valuable associative insights, they fail to elucidate temporal dynamics and mechanistic pathways underlying stress-induced reproductive dysregulation. This limitation is further compounded by heterogeneity in assessment tools, encompassing biochemical assays, psychometric scales, and self-reported measures, thereby introducing variability and reducing reproducibility across studies. Additionally, microbiome research in this domain remains largely correlative, with insufficient mechanistic elucidation of specific microbial taxa and their functional contributions to reproductive endocrinology. The lack of controlled experimental models and interventional trials hamper the identification of causal microbial signatures and their therapeutic potential (59–61). In review another critical dimension that warrants rigorous investigation is the role of sex-specific differences in neuroendocrine–microbiome crosstalk. Emerging studies suggest that hormonal fluctuations, particularly involving oestrogen and progesterone, may differentially modulate gut microbial composition and immune responses, thereby influencing reproductive outcomes. However, these interactions remain inadequately characterised, necessitating targeted, sex stratified research frameworks. Moreover, inter-individual variability in genetic architecture, endocrine profiles, stress responsivity, and microbial ecology underscores the limitations of a "one-size-fits-all" therapeutic paradigm. Future research must adopt longitudinal, multi-omics methodologies integrating genomics, metabolomics, and microbiomics to achieve a systems-level understanding of the gut–brain ovary axis. Such integrative approaches would facilitate the identification of composite biomarker signatures and enable the stratification of patients based on biological and psychosocial phenotypes. Importantly, well-designed randomised controlled trials (RCTs) with robust sample sizes and standardised protocols are imperative to validate emerging therapeutic interventions (62–64). These should encompass multimodal strategies, including CBT, lifestyle modification such as diet, exercise, and sleep optimisation. Additionally, microbiome-targeted therapies like probiotics, prebiotics, and faecal microbiota transplantation found to be extremely result oriented (65–67).

## 10 Strengths and limitations

One of the strengths of this narrative review is the focus on the role of psychological stress, neuroendocrine regulation, microbiome-related pathways, inflammation, and relationship between ovarian function and the stress–gut–ovary framework. The review also correlates the supporting mechanistically with clinically relevant problems such as PCOS, endometriosis, infertility and assisted reproduction. The story of the game however, may have a selection and interpretation bias. The studies included have varying populations, stress measures, methods for sampling the microbiome, methods for assessing reproductive outcomes, and methods of analysis, therefore making it difficult to directly compare the included studies. Many of the findings are from observation or preclinical studies so conclusions can’t be drawn for cause. Other factors that can affect the findings from the microbiome include the phase of the menstrual cycle, ethnicity, contamination of the sample, specific sequencing methods, diet, BMI, and age. Therefore, microbiome interventions should be viewed as promising methods of research, but not as treatment options at this time.

## 11 Conclusion

According to this narrative review, reproductive health of women may be affected by psychological stress through multiple, interrelated neuroendocrine, microbial, metabolic, and immune-inflammatory mechanisms. Chronic hyperactivity of the HPA axis and higher levels of cortisol can disrupt HPG-axis signalling, GnRH pulsatility, gonadotropin production, follicular development and maturation, ovulation and oocyte competence. Concurrently, stress induced changes in gut physiology and microbial environment may lead to enhanced gut permeability, changes in microbial metabolism, systemic inflammation and even changes in oestrogen metabolism by estrobolome.

The mechanisms offer a biologically plausible explanation of the relationships between psychological stress, menstrual disturbance, PCOS, endometriosis, infertility, and assisted reproduction. However, the bias of observational studies, methodological diversity and the possible reverse causality make it difficult to draw definitive causal conclusions, and there is only limited functional microbiome evidence. The idea that the relationship between stress and the estrobolome is particularly promising, but would benefit from a simultaneous assessment of stress markers, microbial enzymatic activity, oestrogen metabolites and reproductive metrics.

Psychological wellbeing should be considered as part of reproductive health and it is important for clinical care to be aware of this, but climate interventions that aim to modify the microbiome should not be implemented in a hasty way before being validated. Established reproductive treatments may be supplemented by psychological support, stress management, healthy dietary patterns, regular exercise and proper sleep regimen. Longitudinal studies and appropriately designed clinical trials combining measurements of hormones, psychological factors, inflammation, microbiomic changes and metabolomic measurements are necessary to determine causality, and to create safe personalised approaches to the gut–brain–ovary axis.
